# Arneth Blood Count in Tuberculosis

**Published:** 1913-11

**Authors:** 


Arneth Blood Count in Tuberculosis. R. S. Cummings of Los Angeles, *Calif. State Jour. of Med.*, July, 1913. In 1896, A. M. Holmes noted a tendency to a disproportionate number of leucocytes containing few nuclei, in tuberculosis. In 1904, Arneth established the following normal standards: 1-nucleus in neutrophiles, 5 per cent.; 2-5 nuclei, respectively, 35 per cent, 41 per cent., 17 per cent., 2 per cent. In tuberculosis, according to the severity of infection, there was a tendency toward disproportionate percentages of cells with few nuclei. For example, 12 per cent., 48 per cent., 30 per cent., 1 per cent., 0 per cent., according as the nuclei numbered 1, 2, 3, 4 and 5 in a cell. Bushnell and Treuholtz in 1908, divided the cells mesially into 1 and 2-nucleated and half of the 3-nucleated, and half the 3-nucleated, 4 and 5-nucleated. Normally 67 per cent. of the neutrophiles should fall in the lower group, while in tuberculosis, 85-90 per cent. would contain few-nucleated cells. In a total of 80 cases on whom 206 blood counts were made, in normal individuals (22 cases, 36 counts) the index ranged 43-66, average 55; in second stage patients (54 cases, 124 counts) 33-81.5, average 60; in third stage patients (26 cases, 82 counts) 52.5-92, average 73.

